# Supplementary material for: Controlling Nutritional Status (CONUT) Score and Sarcopenia as Mutually Independent Prognostic Biomarkers in Advanced Urothelial Carcinoma
Source: Cancers (Basel). 2022 Oct 17;14(20):5075. doi: 10.3390/cancers14205075 (PMC9600715; doi:10.3390/cancers14205075)
Supplement: Supplementary file 1 [file cancers-14-05075-s001.zip › cancers-1905998-supplementary.pptx]

## Slide 1
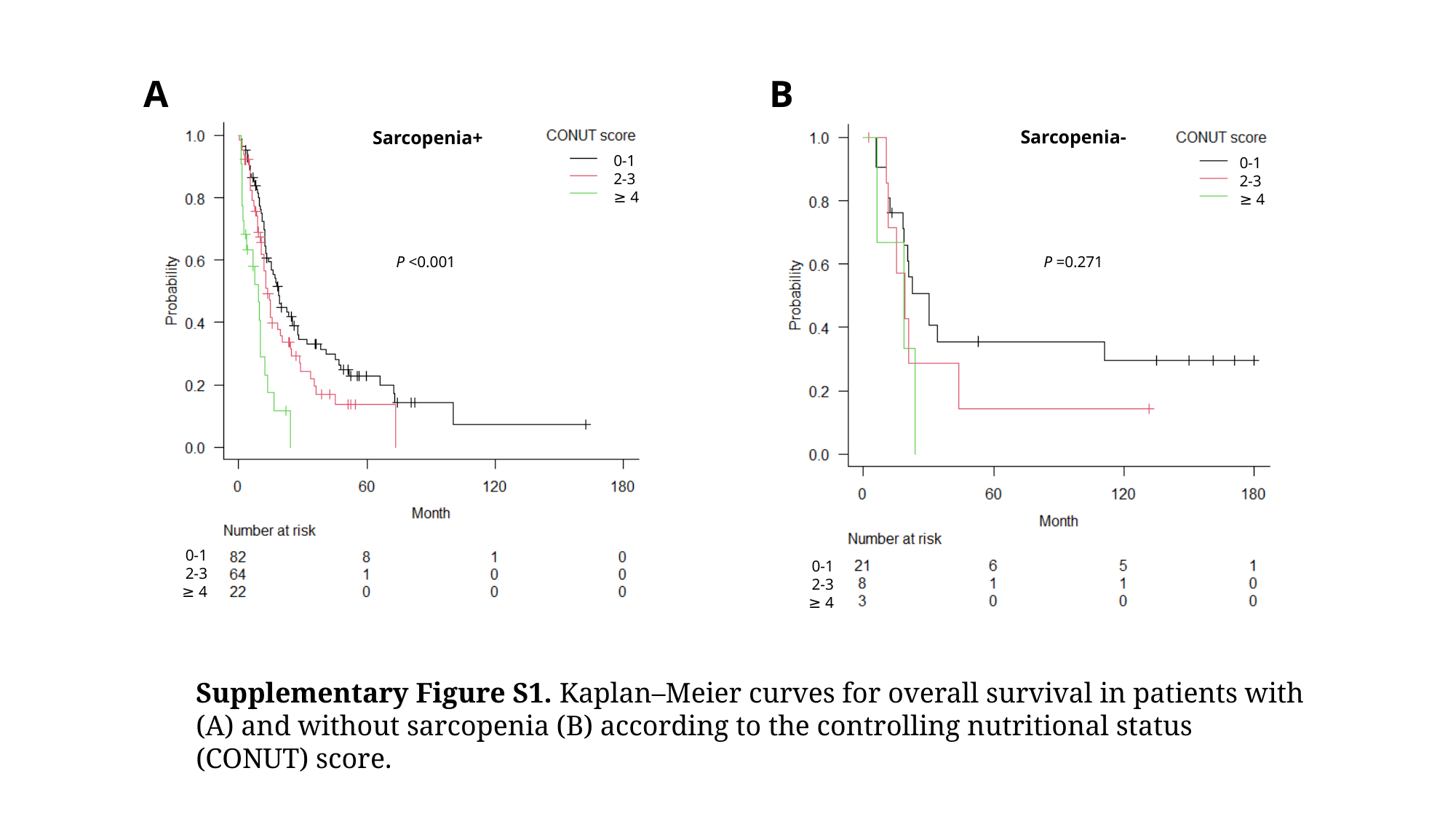

A
B
Sarcopenia-
Sarcopenia+
0-1
2-3
≥ 4
0-1
2-3
≥ 4
P <0.001
P =0.271
0-1
2-3
≥ 4
0-1
2-3
≥ 4
Supplementary Figure S1. Kaplan–Meier curves for overall survival in patients with (A) and without sarcopenia (B) according to the controlling nutritional status (CONUT) score.

## Slide 2
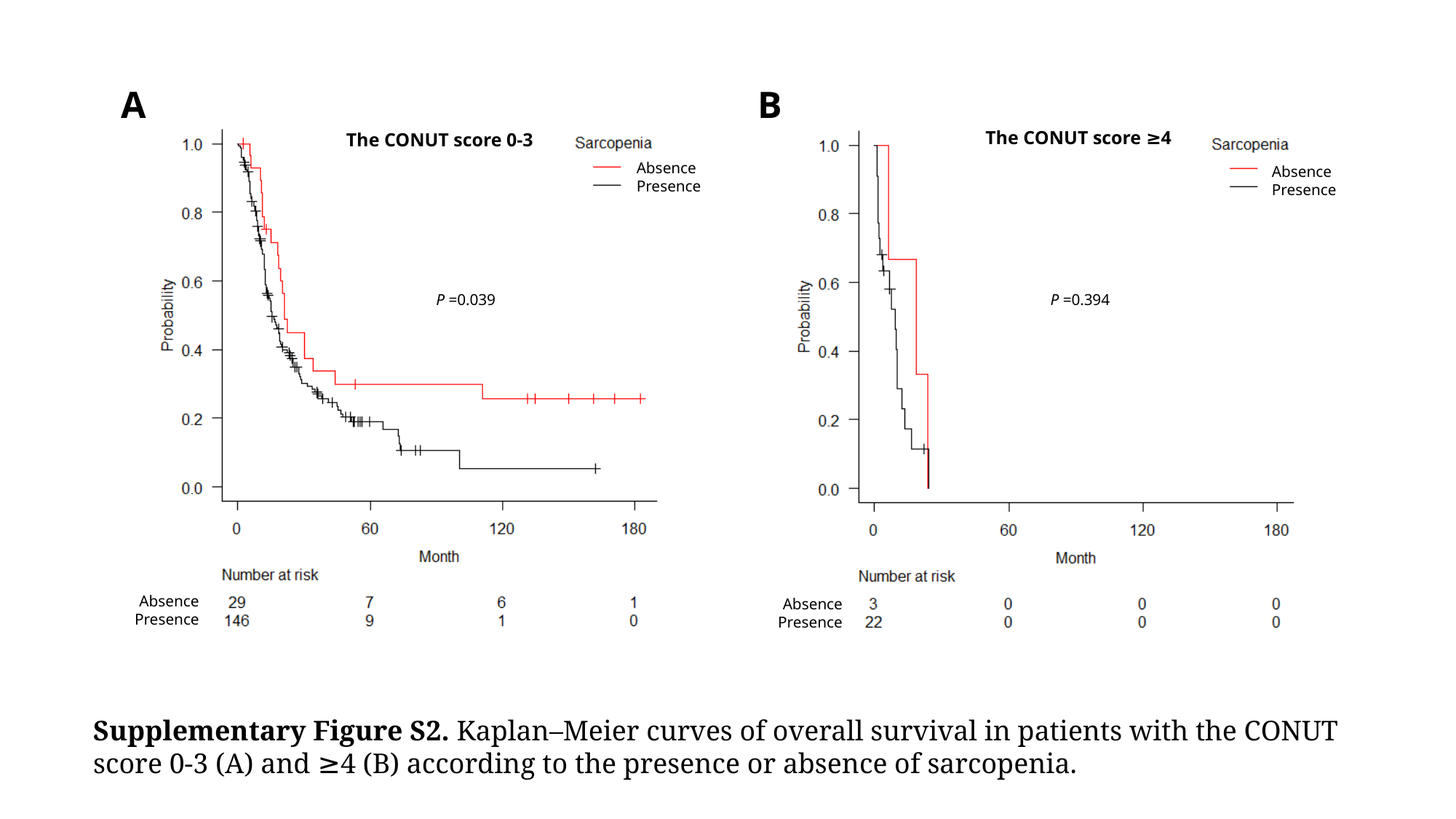

A
B
The CONUT score ≥4
The CONUT score 0-3
Absence
Presence
Absence
Presence
P =0.039
P =0.394
Absence
Presence
Absence
Presence
Supplementary Figure S2. Kaplan–Meier curves of overall survival in patients with the CONUT score 0-3 (A) and ≥4 (B) according to the presence or absence of sarcopenia.
